# Functioning glucagonoma associated with primary hyperparathyroidism: multiple endocrine neoplasia type 1 or incidental association?

**DOI:** 10.1186/1471-2407-12-614

**Published:** 2012-12-22

**Authors:** Enrico Erdas, Nicola Aste, Luca Pilloni, Angelo Nicolosi, Sergio Licheri, Antonello Cappai, Marco Mastinu, Filomena Cetani, Elena Pardi, Stefano Mariotti, Mariano Pomata

**Affiliations:** 1 General Surgery Unit, Department of Surgical Sciences, San Giovanni di Dio Hospital, University of Cagliari, Cagliari, Italy; 2 Dermatology Unit, Department of Medical Sciences, San Giovanni di Dio Hospital, University of Cagliari, Cagliari, Italy; 3 Pathology Unit, Department of Surgical Sciences, San Giovanni di Dio Hospital, University of Cagliari, Cagliari, Italy; 4 Endocrine Surgery Unit, Department of Surgical Sciences, Policlinico di Monserrato, University of Cagliari, Cagliari, Italy; 5 Endocrinology Unit, Department of Medical Sciences, Policlinico di Monserrato, University of Cagliari, Cagliari, Italy; 6 Endocrinology Unit, Department of Internal and Experimental Medicine, University of Pisa, Pisa, Italy

**Keywords:** Multiple endocrine neoplasia type 1, Glucagonoma, Primary hyperparathyroidism

## Abstract

**Background:**

Diagnosis of multiple endocrine neoplasia type 1 (MEN1) is commonly based on clinical criteria, and confirmed by genetic testing. In patients without known MEN1-related germline mutations, the possibility of a casual association between two or more endocrine tumors cannot be excluded and subsequent management may be difficult to plan. We describe a very uncommon case of functioning glucagonoma associated with primary hyperparathyroidism (pHPT) in which genetic testing failed to detect germline mutations of *MEN-1* and other known genes responsible for MEN1.

**Case presentation:**

The patient, a 65-year old woman, had been suffering for more than 1 year from weakness, progressive weight loss, angular cheilitis, glossitis and, more recently, skin rashes on the perineum, perioral skin and groin folds. After multidisciplinary investigations, functioning glucagonoma and asymptomatic pHPT were diagnosed and, since family history was negative, sporadic MEN1 was suspected. However, genetic testing revealed neither *MEN-1* nor other gene mutations responsible for rarer cases of MEN1 (*CDKN1B*/p27 and other cyclin-dependent kinase inhibitor genes *CDKN1A*/p15, *CDKN2C*/p18, *CDKN2B*/p21). The patient underwent distal splenopancreatectomy and at the 4-month follow-up she showed complete remission of symptoms. Six months later, a thyroid nodule, suspected to be a malignant neoplasia, and two hyperfunctioning parathyroid glands were detected respectively by ultrasound with fine needle aspiration cytology and ^99m^Tc-sestamibi scan with SPECT acquisition. Total thyroidectomy was performed, whereas selective parathyroidectomy was preferred to a more extensive procedure because the diagnosis of MEN1 was not supported by genetic analysis and intraoperative intact parathyroid hormone had revealed “adenoma-like” kinetics after the second parathyroid resection. Thirty-nine and 25 months after respectively the first and the second operation, the patient is well and shows no signs or symptoms of recurrence.

**Conclusions:**

Despite well-defined diagnostic criteria and guidelines, diagnosis of MEN1 can still be challenging. When diagnosis is doubtful, appropriate management may be difficult to establish.

## Background

Multiple endocrine neoplasia type 1 (MEN1) is a rare inherited autosomal dominant syndrome characterized by variable combinations of primary hyperparathyroidism (pHPT) (approximately 95% penetrance), pancreatic endocrine tumors (PETs) (40-70% penetrance), and anterior pituitary tumors (30-40% penetrance) [1]. The main causative gene of MEN1 (*MEN-1*) is located at chromosome 11q13 and, during the first decade following its identification, over 1100 germline mutations were discovered [2]. Recently, other germline mutations involving four cyclin-dependent kinase inhibitor genes (*CDKN1A*/p15, *CDKN2C*/p18, *CDKN2B*/p21 and *CDKN1B*/p27) and, in patients with pituitary tumors, the *AIP* gene have been found in a minority of patients with clear MEN1 phenotype [3-5].

According to the current guidelines, individuals with at least two of the three major MEN1 endocrine tumors should be considered to be affected by the MEN1 syndrome [1]. Diagnosis should be confirmed by genetic testing, although a substantial minority of patients (up to 40-50% of those without family history) may not harbor any known gene mutations [1,3-7]. In these cases the possibility of a casual association between two endocrine tumors or the occurrence of a sporadic endocrine tumor in a MEN1 family member must be considered, since management of patients and their families differs considerably depending on whether the endocrine tumors are sporadic or MEN1-related [8-10].

We report a case of typical functioning glucagonoma associated with pHPT in which genetic testing failed to detect *MEN-1* and other known germline mutations associated with MEN1, and we discuss specific problems encountered during the diagnostic and therapeutic workup.

## Case presentation

**Figure 1:**
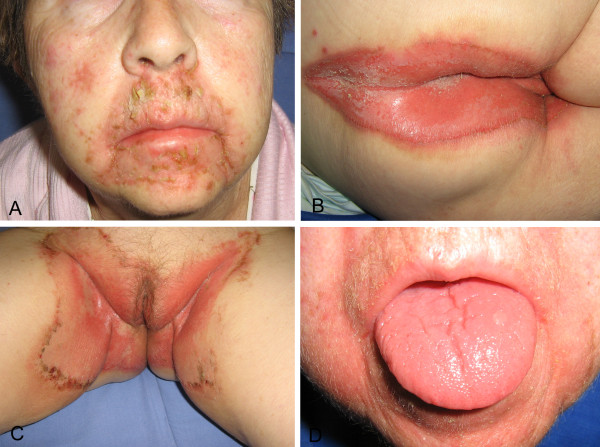
**Skin eruptions.A**) Erythema, scaling, erosions and crusts on the face. **B**) Intense erythema with crusted erosions at perineum. **C**) Polycyclic migratory lesions with scaling advancing borders at groin folds; **D**) Glossitis.

A 65-year-old woman with no family history of endocrine tumors was referred to our General Surgery Unit with a presumptive diagnosis of MEN1. For the past 18 months, she had been experiencing increasing weakness, weight loss (up to 15 kg), angular cheilitis, and glossitis. In the meantime, due to a traumatic fracture of her left humeral head, she had undergone dual energy x-ray absorptiometry and laboratory investigations as an outpatient, which were suggestive of severe osteoporosis (t-score −4 at the lumbar spine and −2.4 at the femoral neck), pHPT, hypothyroid Hashimoto’s thyroiditis, and diabetes mellitus type 2. The patient had recently developed widespread itching and painful rashes involving the perioral skin, perineum, and groin folds (Figure 1). In view of these multiple findings she was admitted to an Internal Medicine Unit for further assessment. Her father had died at age 84 due to myocardial infarction and her mother at age 69 after colorectal cancer surgery. A 60-year-old brother suffered from arterial hypertension, and a 32-year-old daughter was affected by severe obesity. Menarche occurred at 12 years of age and menopause at 39 years following hystero-adnexectomy for post-partum uterine rupture. There were no other remarkable data in her medical history, and she was not taking any drugs. Biochemical studies showed iron-deficiency anemia and confirmed Hashimoto’s thyroiditis with mild hypothyroidism, diabetes mellitus, and mild pHPT (Calcium: 10.4 mg/dl [nr 8.8-10.6], 24-hour urinary calcium excretion: 358 mg/dl [nr 130–300], iPTH: 147pg/ml [nr 8–87], Creatinine: 0.74 mg/dl [nr 0.84-1.25]). On ^99m^Tc-sestamibi scan and ultrasound (US) of the neck, an inferior right hyperfunctioning parathyroid was identified. A 9 mm nodule was also detected by US in the left thyroid lobe. Endoscopic studies revealed mild antral gastritis and diverticulosis of the colon, while no pathological findings were detected by abdominal US. Based on skin culture, the skin rashes were interpreted as candidiasis secondary to Candida albicans with bacterial superinfection. The patient was then discharged with a prescription of oral antidiabetics, iron therapy, proton pump inhibitors, bisphosphonates, levothyroxine and antifungal/antibiotic agents.

**Figure 2:**
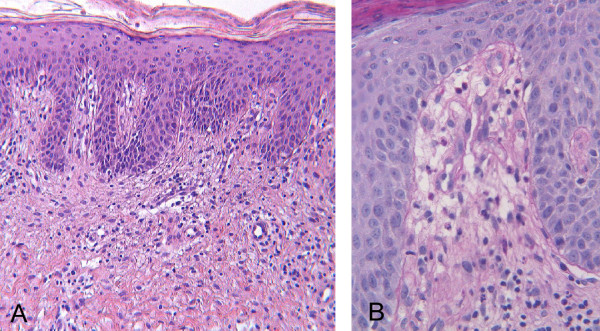
**Histopathological examination of the skin.A**) Psoriasiform hyperplasia of the epidermis with overlying parakeratosis and mild perivascular infiltrate of lymphocytes in the upper dermis (HE 5 X). **B**) Vascular dilatation (HE 20 X).

**Figure 3:**
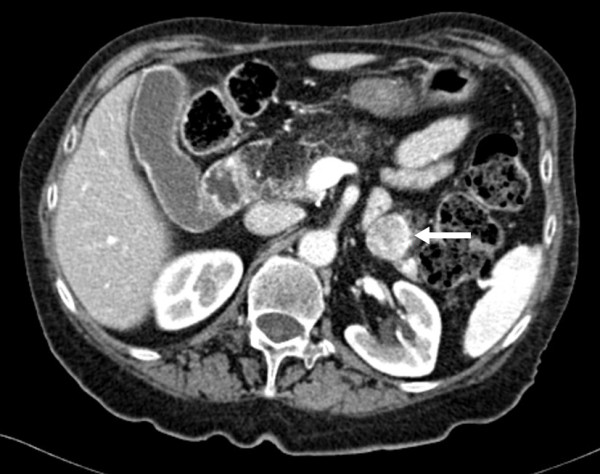
**Abdominal enhanced multidetector-row computed tomography (MDCT).** A low-density 2x3 cm mass between the body and tail of the pancreas, showing intense contrast enhancement (arrow).

**Figure 4:**
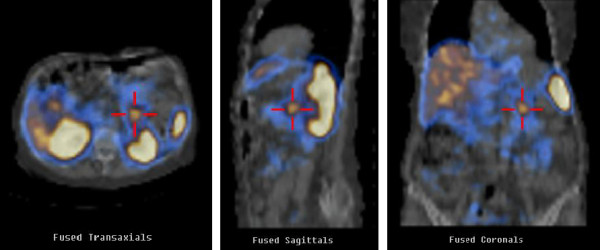
**Somatostatin receptor scintigraphy with SPECT acquisition.** Area of increased uptake anterior to the left kidney and medial to the spleen, consistent with the pancreatic mass detected by MDTC.

After one month, as the rash had not improved the patient was referred to the Dermatology Unit, where a generic deficiency dermatitis was diagnosed based on histological examination of a skin biopsy (Figure 2). Oral zinc and vitamin supplements were introduced into her diet, but no improvement was observed over the following 2 months. Since the histological features of deficiency dermatitis were also consistent with necrolytic migratory erythema (NME), abdominal enhanced multidetector-row computed tomography (MDCT) was performed, revealing a low-density 2x3 cm mass between the body and tail of the pancreas, with intense contrast enhancement, compatible with a diagnosis of neuroendocrine neoplasia (Figure 3). No evidence of liver or lymph node metastasis or local infiltration was found. Therefore the patient was referred to the Endocrinology Unit with suspected glucagonoma syndrome. As glucagon testing was not available, only generic neuroendocrine markers were measured, and among these, only Chromogranin A was found to be above the normal range (urinary 5-Hydroxyindoleacetic acid excretion: 5.8 ng/24h [nr 2–9]; serum Neuron-Specific Enolase: 12 ng/ml [nr 4.7-14.7]; serum Chromogranin A: 24.9 nmol/L [nv <4]. Somatostatin receptor scintigraphy (OctreoScan) with SPECT acquisition showed an area of increased uptake between the body and tail of the pancreas corresponding to the tumor revealed by previous MDCT (Figure 4). Phospho-calcium metabolism assessment confirmed pHPT (Calcium: 11.8 mg/dl [nr 8.8-10.6]; Phosphorus: 2.4 mg/dl [nr 2.5-4.5]; 24-hour urinary calcium excretion: 212 mg/dl [nr 100–300]; iPTH: 120 pg/ml [nr 8–87]). All pituitary hormones were in the normal range and no pituitary tumor was detected by magnetic resonance imaging.

Owing to the joint presence of PET and pPTH and the absence of a family history of single or combined endocrine tumors, a presumptive diagnosis of sporadic MEN1 syndrome was made. Genetic testing was firstly focused on the *MEN-1* gene; for this purpose, exons 2–10 were PCR amplified and subsequently submitted to direct sequencing according to standard protocols [11], but no mutation was identified.

**Figure 5:**
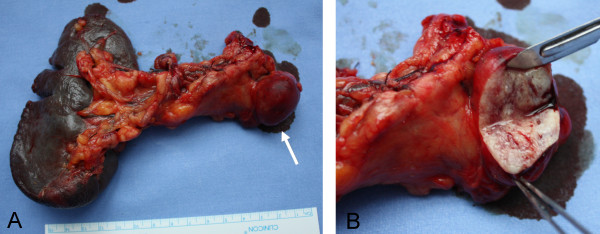
**Specimen from distal splenopancreatectomy.A**) The neoplasia is located in the inferior border of the pancreas (arrow); it shows an exophytic growth but appears well circumscribed. **B**) The cut surface is whitish-yellow in color with focal areas of hemorrhage.

**Figure 6:**
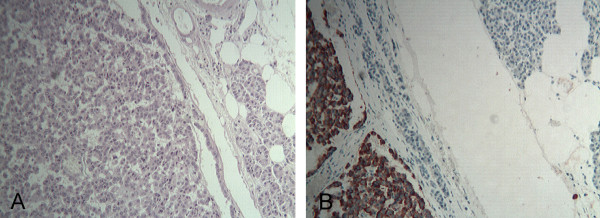
**Histopathological examination of the pancreatic tumor.A**) The tumor appears encapsulated and composed of polygonal cells with trabecular or ribbon-like proliferation (HE 5 X). **B**) At immunohistochemistry, neoplastic cells showed an intense diffuse staining for glucagon (Anti-glucagon antibody 5 X).

The patient was then referred to our General Surgery Unit where she underwent distal splenopancreatectomy. Intraoperative US ruled out tumor multifocality and showed no liver or lymph node metastasis, nor any infiltration of the splenic and superior mesenteric vessels. The postoperative course was uneventful and the patient was discharged 11 days after the operation in good condition. The resected specimen contained a tumor measuring 3×2×2 cm in diameter, corresponding to the lesion identified by imaging studies (Figure 5). On histopathological examination the tumor appeared encapsulated, well vascularized, and composed of polygonal cells with trabecular or ribbon-like proliferation. Four mitoses per 10 HPF (High Power Field) were observed. No lymphatic, blood vessel or perineural invasions were found. Immunohistochemistry showed a 5% Ki-67 index and intense diffuse staining for non-specific neuroendocrine markers (Chromogranin A, Synaptophysin, Neuron-Specific Enolase) and for glucagon (Figure 6), thus confirming the preoperative suspicion of pancreatic glucagonoma.

**Figure 7:**
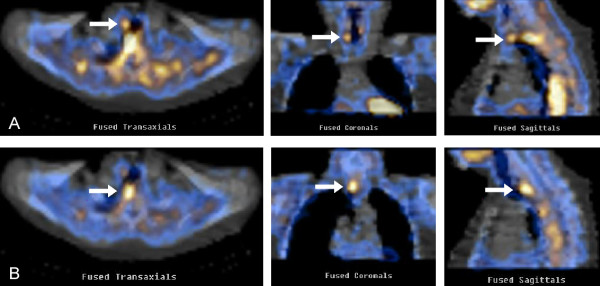
^**99m**^**Tc-sestamibi scan with SPECT acquisition.** Hyperfunctioning parathyroid glands (arrows) detected respectively to the right of (**A**) and behind the trachea (**B**).

**Figure 8:**
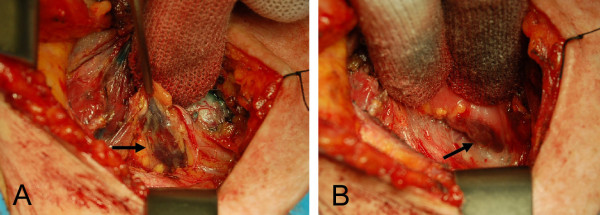
**Intraoperative view of the two enlarged parathyroid glands.** One is located in the right para-tracheal space (arrow) (**A**) and the other is posterior and closely adherent to the esophagus (arrow) (**B**).

During the following 4 months, the patient had complete resolution of her diabetic-dermatogenic syndrome and gained about 10 kg in body weight. At the 1-year follow up, abdominal US and OctreoScan with SPECT acquisition revealed no signs of recurrence, while serum Chromogranin A remained slightly elevated (4.6 nmol/l [nv < 4]). Anterior pituitary hormone levels were normal and parathyroid function showed no significant change from the previous examination. However, a new hyperfunctioning parathyroid gland, in addition to that previously identified, was found behind the trachea on ^99m^Tc-sestamibi scan with SPECT acquisition (Figure 7). US examination of the neck confirmed a 9-mm nodule in the middle third of the left thyroid lobe, but detected no enlarged parathyroid glands. Fine needle aspiration cytology allowed diagnosis of the thyroid nodule as Thy 4 (i.e. suspicious for malignancy), according to the BTA classification [12]. Based on these findings, the patient was referred to the Endocrine Surgery Unit with indications for total thyroidectomy and subtotal parathyroidectomy (PTX). The procedure was performed via Kocher cervicotomy with the help of intraoperative nerve monitoring and iPTH assay. After bilateral neck exploration, two enlarged parathyroid glands were found respectively in the right para-tracheal space and behind the esophagus (Figure 8), as indicated by preoperative scintiscan. At 10 minutes from the first PTX, iPTH was higher than 50% (90 pg/ml) of the initial basal value (176 pg/ml), but fell below that threshold at 20 minutes (49 pg/ml). Ten minutes after the second PTX, iPTH dropped markedly to 32 pg/ml, and therefore it was decided not to resect the remaining two left parathyroid glands, which had a normal appearance. Total thyroidectomy was performed as planned. On histopathological examination, Hürthle cell adenoma of the thyroid and diffuse/nodular parathyroid hyperplasia were diagnosed. The patient was discharged 3 days after surgery in good condition. Calcium and vitamin D supplements were necessary only for a few days. Thirty-nine and 25 months after respectively the first and the second operations, the patient is well and shows no signs or symptoms of recurrence. Since mutations of CDKN complex have been recently described in MEN1 patients with negative *MEN-1* mutations, a genetic study was first carried out on the *CDKN1B*/p27 gene by PCR amplification and direct sequencing of exons 1 and 2 [3,4], but no mutation was found. Analysis of other CDKN complex genes (*CDKN1A*/p15, *CDKN2C*/p18, *CDKN2B*/p21) was then carried out by the same technique [3,4], and again no mutation was found. To exclude large deletions of the *MEN-1* gene which may be missed using the conventional PCR amplification and direct sequencing approach [13,14], multiple ligation-dependent probe amplification (MLPA) analysis [15] was carried out using the Salsa MLPA probemix P244-B1, but no deletions of the *MEN-1* gene were identified. In line with the current indications [6,7], no *AIP* gene mutation search was performed, as the patient had no evidence of pituitary tumors.

Although the MEN1 syndrome had not been confirmed by genetic analysis, based on the new 2012 guidelines for MEN1 [1] we recently proposed to the daughter of the patient that she undergo clinical and biochemical screening, but she has refused for the time being.

## Discussion

According to the current guidelines [1] an individual affected by two or more primary MEN1-related endocrine tumors should be suspected to have the MEN1 syndrome. However, association of such tumors may occur randomly in the general population [9], therefore patients without family background should be candidates for genetic testing in order to confirm the diagnosis [1]. Accordingly, extensive analysis of the *MEN-1* gene (including the search for large deletions by MPLA) and of the CDKN genes was performed, but no mutations were found. AIP analysis was not performed since previous studies indicated that this was not required in patients without pituitary tumors [6,7]. Thus, to the best of our knowledge, the genetic study was in this case complete and up to date, although other conditions (mutations in noncoding regions - e.g., exon 1-, false negative results in direct sequencing, and mutations of other still unknown genes) may cause failure to detect germline mutations. In summary, since DNA test results may be negative in up to 20% of index cases for familial MEN1 and even more frequently in apparently sporadic cases [1-8], the lack of genetic abnormalities does not necessarily rule out a diagnosis of MEN1 when the clinical criteria are met. On the other hand, several additional findings can support a suspected diagnosis, including lesions occurring before the age of 35, multiglandular pHPT, and multiple lesions within the pancreas [9]. Our case met the clinical criteria for MEN1, but extensive genetic testing targeting all the presently known MEN1-related genes was negative, and the clinico-pathological behavior of the associated endocrine lesions provided inconsistent pathogenic information. Glucagonoma syndrome developed when the patient was 63 years old and it was secondary to a single pancreatic tumor. pHPT was discovered by chance, since it was asymptomatic according to NIH criteria [16], and multiglandular hyperplasia was found at histological examination. The late onset and the absence of multifocality are suggestive for non-familial glucagonoma, and the association with pHPT may have been casual since it occurs in over 2% of adults aged over 55 [17]. Nevertheless, MEN1 can affect almost all age groups, with a reported age range of 5–81 years [1,8,18] and glucagonoma may not show multifocal appearance in over 40% of MEN1 patients [19]. pHPT occurs very early in MEN1 patients, typically between 20 and 25 years of age, and precedes the appearance of the other endocrine disorders by as much as a decade [20]. Unfortunately, as in this case pHPT was discovered by chance, it is impossible to establish the age of onset. The finding of multiple parathyroid disease matches favorably with the diagnosis of MEN1, although it must be noted that most patients with multiglandular hyperplasia do not have familial pHPT [21]. Taken together, the above data neither support nor exclude a diagnosis of MEN1.

Glucagonoma is a very uncommon PET, with an estimated incidence of 0.05-0.1/1.000.000 [22,23]. It generally develops as a sporadic (i.e. non-familial) neoplasm, and in 51-78% of cases it is associated with metastasis at the time of diagnosis [19,22]. The rate of metastasis is directly proportional to tumor size, being more than 47% when the tumor is 21–50 mm in diameter [19]. In only 5-17% of cases does glucagonoma occur in the context of MEN1, and for this reason data on its biological and clinical behavior in this setting are poor and inconsistent [9,19,24-26]. In a large review of glucagonomas, malignancy is reported to be lower in patients with MEN1 than in those without it (24.5% vs 66.1%) [19]. By contrast, two recent studies by GTE (Groupe d’etude des Tumours Endocrines) have shown a low 10-year survival rate (53,8%) and a high disease-specific risk of death (hazard ratio 4.29) in MEN1 patients, although the data are not referred specifically to glucagonomas, but rather to a miscellaneous group of rare PETs including VIPomas and somatostatinomas [9,26]. In our case, the glucagonoma must be considered as an intermediate grade neuroendocrine tumor (NET G2) or as stage 1B (T2N0M0) according respectively to the WHO 2010 classification [27] and the AJCC TNM staging (7^th^ ed., 2010) [28]. It was functioning and gave rise to a typical cluster of symptoms including anemia, weight loss, asthenia, diabetes and NME. Fortunately, the treatment of glucagonoma does not differ significantly between MEN1 and sporadic cases [29,30], therefore the operation we performed, i.e. distal splenopancreatectomy, can be considered appropriate.

As regards the pHPT, although it was asymptomatic, indication for surgery was established firstly because the patient had to undergo total thyroidectomy for a concomitant suspicion of thyroid carcinoma, and secondly because she suffered from severe osteoporosis. One could argue that, according to NIH criteria [16], the T-score at the femoral neck was slightly above the threshold value (−2.4 vs −2.5), and that bone mineral density should have been evaluated also at the forearm. However, the AACE/AAES task force on pHPT has recently stated that operative management should be considered for all asymptomatic patients with suitable risk factors and a reasonable life expectancy [17]. The reason for this recommendation is that 23-62% of asymptomatic patients develop symptoms or complication at 10 years, and patients with untreated pHPT have an increased risk of premature death from cardiovascular diseases and malignant lesions. Furthermore, many symptoms not addressed by NIH guidelines, such as weakness, apathy, depression, malaise, mood swings, sleep disorders and impaired mental clarity usually decrease after successful PTX even in patients with very mild disease [17,31]. All things considered, our indication for PTX may be considered to be correct, whereas the choice of surgical procedure may be questioned. There is widespread consensus that subtotal or total PTX are the most suitable treatments for pHPT in MEN1 patients, since more conservative surgery is associated with a very high rate of persistent or recurrent disease [21,29]. What is still debated is which of the two is the safest and most effective procedure, given that subtotal PTX is associated with a higher frequency of recurrent pHPT, but total PTX gives rise more commonly to persistent hypocalcaemia [1,20,21,32]. In our case, the final decision to perform selective PTX was based on at least three reasons: a lack of genetic confirmation of MEN1, the presence of only 2 enlarged parathyroid glands at neck exploration, and a rapid fall in intraoperative iPTH levels after the second PTX. Twenty-five months after surgery the patient shows no signs of persistent or recurrent pPTH, although this result needs to be confirmed by longer-term follow-up. Other authors have achieved optimal outcomes following a similar surgical strategy. Lee et al. reported no recurrence with more than five years’ follow up in seven MEN1 patients affected by pHPT who underwent selective PTX [33]. Kraimps et al. found that recurrent hyperparathyroidism occurred more frequently after subtotal PTX in patients with diffuse hyperplasia than after selective PTX in patients with one or two enlarged parathyroid glands [34]. Tonelli et al., while considering total PTX as the standard treatment for pHPT in MEN1, performed conservative surgery on one patient showing “adenoma-like” kinetics of intraoperative iPTH, and at the 4-year follow-up they did not detect recurrent disease [35]. Overall, these authors believe that a less aggressive MEN1 variant may exist. Therefore, in selected cases, such as lack of genetic preoperative diagnosis of MEN1, evidence of less than four enlarged parathyroids, and “adenoma-like” kinetics of intraoperative iPTH, they believe conservative PTX should be taken into consideration [33-35].

## Conclusions

The case presented here illustrates the fact that diagnosing MEN1 can remain difficult despite the giant strides made in diagnostic imaging and genetic research. Where, as in this case, strict adherence to the current guidelines is not feasible, appropriate management may be difficult to establish.

### Consent

Written informed consent was obtained from the patient (March 2012) for publication of this case report and any accompanying images. A copy of the written consent is available for review by the Editor-in-Chief of this journal.

## Abbreviations

MEN 1: Multiple endocrine neoplasia type 1; pHPT: Primary hyperparathyroidism; PETs: Pancreatic endocrine tumors; CDKN: Cyclin-dependent kinase inhibitor genes; AIP: Aryl hydrocarbon receptor Interacting Protein; nr: Normal range; iPTH: Intact parathyroid hormone; US: Ultrasound; NME: Necrolytic migratory erythema; MDCT: Multidetector-row computed tomography; nv: Normal value; HPF: High Power Field; WHO: World Health Organization; AJCC: American Joint Committee on Cancer; BTA: British Thyroid Association; PTX: Parathyroidectomy; GTE: Groupe d’etude des Tumours Endocrines; NIH: National Institute of Health; AACE/AAES: American Association of Clinical Endocrinologists/American Association of Endocrine Surgeons; PCR: Polymerase chain reaction; MLPA: Multiplex ligation-dependent probe amplification.

## Competing interests

The authors declare that they have no competing interests.

## Authors’ contributions

EE conceived the study, participated in its design and coordination, and drafted the manuscript. NA, SL and MP participated in the study design and helped to draft the manuscript. LP performed the histopathological stainings, took all micrographs and participated in the drafting of the manuscript. AN participated in the study design and in critical revision of the manuscript. AC, FC and EP participated in the study design and performed the genetic studies and sequence analysis. MM participated in study design and coordination in clinical data acquisition. SM participated in drafting of the manuscript and its critical revision. All authors read and approved the final manuscript.

## Pre-publication history

The pre-publication history for this paper can be accessed here:

http://www.biomedcentral.com/1471-2407/12/614/prepub
